# *Imidazopyranotacrines* as Non-Hepatotoxic, Selective Acetylcholinesterase Inhibitors, and Antioxidant Agents for Alzheimer′s Disease Therapy

**DOI:** 10.3390/molecules21040400

**Published:** 2016-03-24

**Authors:** Houssem Boulebd, Lhassane Ismaili, Manuela Bartolini, Abdelmalek Bouraiou, Vincenza Andrisano, Helene Martin, Alexandre Bonet, Ignacio Moraleda, Isabel Iriepa, Mourad Chioua, Ali Belfaitah, José Marco-Contelles

**Affiliations:** 1 Equipe de Synthèse de Molécules à Objectif Thérapeutique, Laboratoire des Produits Naturels d′Origine Végétale et de Synthèse Organique (PHYSYNOR), Université des frères Mentouri, Campus de Chaabat-Ersas, Constantine 25000, Algeria; boulebdhoussam@hotmail.fr (H.B.); bouraiou.abdelmalek@yahoo.fr (A.B.); 2 Laboratoire de Chimie Organique et Thérapeutique, Neurosciences Intégratives et Cliniques EA 481, UFR SMP, Univ. Franche-Comté, Univ. Bourgogne Franche-Comté, 19, rue Ambroise Paré, F-Besançon 25000, France; 3 Department of Pharmacy and Biotechnology, University of Bologna, Via Belmeloro 6, Bologna 40126, Italy; manuela.bartolini3@unibo.it; 4 Department for Life Quality Studies, University of Bologna, Corso d′Augusto, 237, Rimini 47921, Italy; vincenza.andrisano@unibo.it; 5 Laboratoire de Toxicologie Cellulaire, EA 4267, Univ. Bourgogne Franche-Comté, 19, rue Ambroise Paré, Besançon Cedex 25030, France; helene.martin@univ-fcomte.fr (H.M.); alexandre.bonet@univ-fcomte.fr (A.B.); 6 Departamento de Química Orgánica y Química Inorgánica, Facultad de Biología, Ciencias Ambientales y Química, Universidad de Alcalá, Ctra. Barcelona, Km. 33.5, Alcalá de Henares 28817, Spain; ignacio.moraleda@uah.es (I.M.); isabel.iriepa@uah.es (I.I.); 7 Laboratory of Medicinal Chemistry (IQOG, CSIC), C/Juan de la Cierva 3, Madrid 28006, Spain; mchioua@gmail.com

**Keywords:** tacrine analogues, hepatotoxicity, cholinesterase inhibitors, antioxidant activity, ORAC, Alzheimer′s disease

## Abstract

Herein we describe the synthesis and *in vitro* biological evaluation of thirteen new, racemic, diversely functionalized *imidazo pyranotacrines* as non-hepatotoxic, multipotent tacrine analogues. Among these compounds, 1-(5-amino-2-methyl-4-(1-methyl-1*H*-imidazol-2-yl)-6,7,8,9-tetrahydro-4*H*-pyrano[2,3-*b*]quinolin-3-yl)ethan-1-one (**4**) is non-hepatotoxic (cell viability assay on HepG2 cells), a selective but moderately potent *Ee*AChE inhibitor (IC_50_ = 38.7 ± 1.7 μM), and a very potent antioxidant agent on the basis of the ORAC test (2.31 ± 0.29 μmol·Trolox/μmol compound).

## 1. Introduction

Alzheimer′s disease (AD) is an age-related neurodegenerative disorder whose prevalence is expected to rise significantly in the next decades as the average age of the population increases [1]. Worldwide, around 40 million people suffer from AD [2], which is characterized by a progressive memory loss, a decline in language skills and motor capabilities along with other cognitive impairments [3]. Although the etiology of AD is not completely known, common hallmarks such as β-amyloid (Aβ) deposits [4], τ-protein aggregation [5] and oxidative stress [6] play key roles in the physiopathology of the disease [7]. In addition, the selective loss of cholinergic neurons in AD results in a deficit of the neurotransmitter acetylcholine (ACh) in specific brain regions that mediate learning and memory [8]. Alterations in the serotoninergic and dopaminergic systems are also thought to be responsible for the behavioral disturbances observed in AD patients [9]. At present, there are three FDA-approved drugs (donepezil, galantamine and rivastigmine) [10] that ameliorate AD symptoms by inhibiting acetylcholinesterase (AChE), the enzyme responsible for the hydrolysis of ACh, and by rising the ACh content in the synapsis [11]. Apart from the beneficial palliative properties of AChE inhibitors (AChEI) in AD, cholinergic drugs have shown little efficacy to prevent the progression of the disease. Consequently, nowadays there is no efficient therapy to cure, stop or even slow the progression of the disease. Hence, effective therapeutics are urgently sought [12].

The failure to find such a drug or treatment is possibly due to the multifactorial nature of AD. Thus, single drugs acting on specific targets to produce the expected clinical effects might not be effective in interfering with the complex nature of AD. This is the reason why the multi-target-directed ligand (MTDL) approach [13] has received an increasing attention by many research groups that have developed a variety of compounds binding very diverse biochemical targets [14].

**Figure 1:**
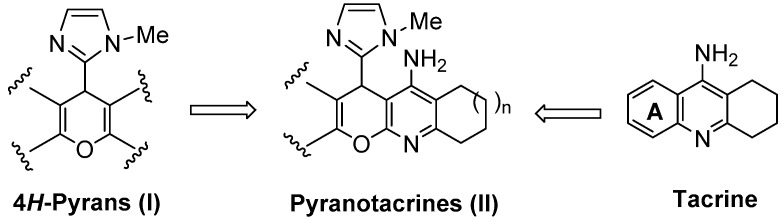
General structure of tacrine, 4*H*-pyrans (**I**), and the new racemic *imidazopyranotacrines* (**II**).

Among the AChEIs investigated in the search for a pharmacological treatment for AD, the case of tacrine is particularly relevant [15]. Tacrine (Figure 1) is one of the strongest butyrylcholinesterase (BuChE) inhibitors known to date [16]. This drug, which was withdrawn from the clinical use for AD treatment because of significant liver toxicity [17], has been largely stigmatized instead of being further developed looking for other non-toxic tacrines retaining valuable anti-ChE properties [18]. In this context, we have reconsidered tacrine as a starting scaffold in the design of new Multipotent Non-Toxic Tacrines (MNTTs), [19] by incorporating other pharmacophoric groups capable of binding or inhibiting other molecular targets involved in the onset and/or progression of the disease. With this idea in mind, since 1997 we developed different types of structurally simple and easily available MNTTs [20].

Thus, expanding our contribution in this area, in this work we report the synthesis of a number of new *imidazopyranotacrines* (**II**), a new family of tacrine derivatives resulting from the substitution of the ring A in tacrine by the 4*H*-pyrano (**I**) heterocyclic motif (Figure 1). In this case, we have incorporated, and at the same position, instead of a substituted phenyl ring [21], or a five- (furan [22], thiophen [22], pyrrole [22]) or six-membered heterocyclic ring system [23], the five-membered 1-methyl-1*H*-imidazol-2-yl motif, in order to improve previous biological results and gain new insights on the structure-activity relationship (SAR) of pyranotacrines. The imizadole ring is a privileged heterocyclic ring system [24] which is present in a number of interesting biologically active molecules such as saripidem, necopidem or zolpidem [25]. This choice was also favored by the ready availability of the imidazole substituted starting derivatives.

**Figure 2:**
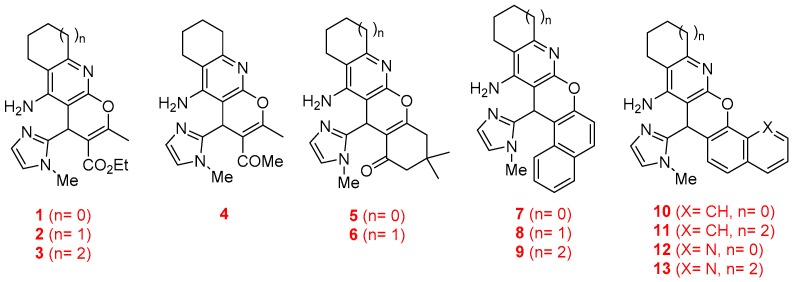
Structures of the *imidazopyranotacrines* **1**–**13**.

Thus, in this work, we describe the synthesis and biological evaluation of the new *imidazopyranotacrines* **1**–**13** (Figure 2), the inhibition of AChE and BuChE enzymes, the antioxidant activities (ORAC-FL method), as well as the evaluation of their hepatotoxicity on HepG2 cells. As a result, we have identified racemic 1-(5-amino-2-methyl-4-(1-methyl-1*H*-imidazol-2-yl)-6,7,8,9-tetrahydro-4*H*-pyrano[2,3-*b*]quinolin-3-yl)ethan-1-one (**4**) (Figure 2) as a non-hepatotoxic compound on HepG2 cells, endowed with selective but moderate inhibitory activity against AChE from *Electrophorus electricus* (*Ee*AChE), and very potent antioxidant activity.

## 2. Results and Discussion

### 2.1. Chemistry

**Scheme 1:**
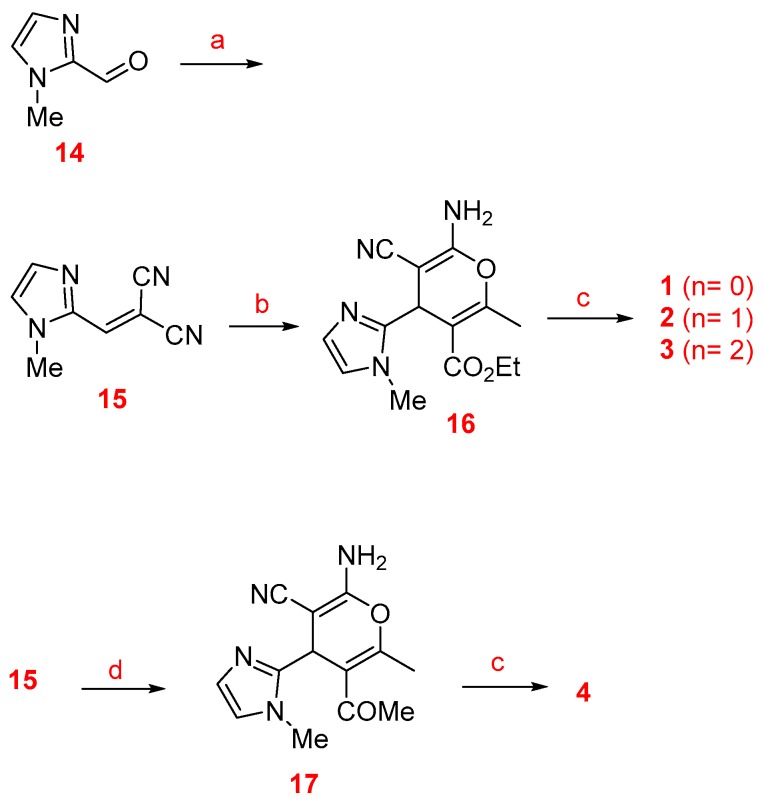

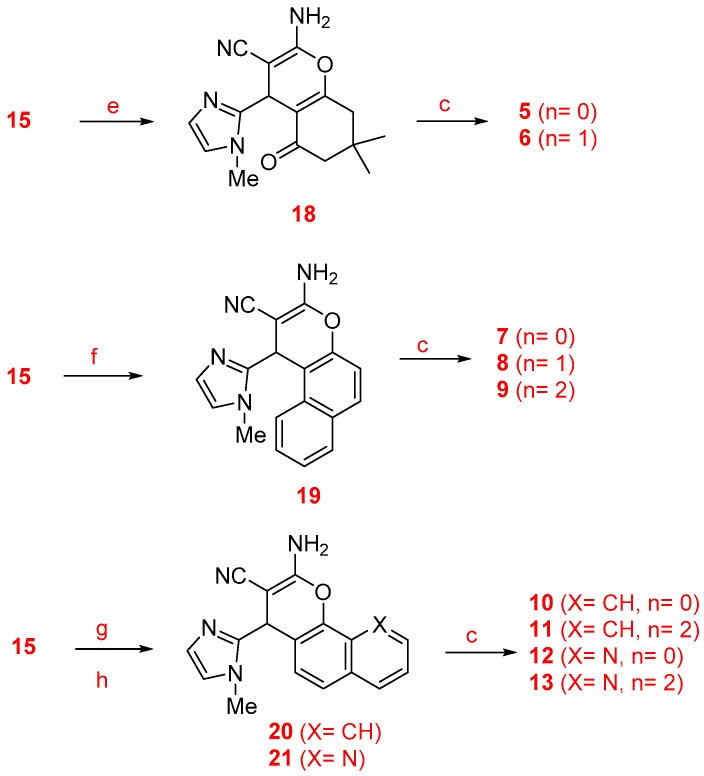
Synthesis of the *imidazopyranotacrines* **1**–**13**. *Reagents and conditions*: (a) Malononitrile, piperidine, ethanol, rt (93%); (b) ethyl 3-oxobutanoate, piperidine, ethanol, rt (92%); (c) cyclopentanone (*n* = 0), cyclohexanone (*n* = 1), cycloheptanone (*n* = 2), AlCl_3_, 1,2-dichloroethane, reflux; (d) pentane-2,4-dione, piperidine, ethanol, rt (90%); (e) 5,5-dimethylcyclohexane-1,3-dione, piperidine, ethanol, rt (69%); (f) 2-naphthol, piperidine, ethanol, rt (72%); (g) 1-naphthol, piperidine, ethanol, rt (75%); (h) 8-hydroxyquinoline, piperidine, ethanol, rt (47%).

The synthesis of racemic *imidazopyranotacrines* **1**–**13** has been carried out by Friedländer-type reactions [26], by treating readily available 4*H*-pyrans with commercial cycloalkanones, under standard reaction conditions, and in good chemical yields. Cyclopentanone, cyclohexanone and cycloheptanone were chosen in order to evaluate the effect of the size of the cycloalkyl ring on the biological activity of the new pyranotacrines (Scheme 1). Starting from 1-methyl-1*H*-imidazole-2-carbaldehyde (**14**), we synthesized 2-(1-methyl-1*H*-imidazol-2yl)methylene)malononitrile (**15**), whose reaction with the appropriate 1,3-dicarbonylic compound or naphthol derivative, gave the key 4*H*-pyrans. For the synthesis of pyranotacrines **1**–**3**, we have used ethyl acetoacetate as starting material, thus leading to *imidazopyranotacrines* bearing a methyl and ethoxycarbonyl substituents at C-2 and C-3, respectively. Similarly, for the synthesis of the pyranotacrines **4** and **5** or **6**, we used 2,4-pentandione, and 5,5-dimethylcyclohexane-1,3-dione, respectively. Finally, for the synthesis of the remaining pyrano-tacrines **19**–**21** we have prepared some conveniently key functionalized 4*H*-pyrans from 1-naphthol, 2-naphthol, and 8-hydroxyquinoline. All new compounds showed excellent analytical and spectroscopic data, in good agreement with their structures.

### 2.2. Biological Assays

#### 2.2.1. Inhibition of the Cholinesterase Enzymes and Kinetic Analysis

First of all, we addressed the capacity of *imidazopyranotacrines* **1**–**13** to inhibit *Ee*AChE and serum horse BuChE (*eq*BuChE) by using Ellman′s assay [27], and tacrine as reference compound for comparative purposes [28].

As shown in Table 1, racemic *imidazopyranotacrines* **1**–**13** were very modest *Ee*AChE inhibitors with potencies in the micromolar range, resulting from two to three orders of magnitude less active than the reference compound tacrine. On the other hand, all derivatives acted as highly selective AChEI, as no or very moderate inhibition of *eq*BuChE activity was observed. The most potent *Ee*AChEI was the *imidazopyranotacrine* **8** (IC_50_ = 6.73 ± 0.52 µM), followed, in decreasing order of activity, by **7**, **10**, **2** and **1**. The three most potent *Ee*AChEIs among the *imidazopyranotacrines* (*i.e.*, **8**, **7** and **10**) bear a naphthol ring fused to the central 4*H*-pyran motif. Furthermore, the 2-naphthol derivatives (**7**, **8**) were more potent than the 1-naphthol ones (**10**, **11**). The 2-naphthol derivative bearing a cyclohexane ring (**8**) is more potent than the analogues bearing a cyclopentane motif (**7**) or a cycloheptane motif (**9**). Note that the incorporation of a heterocyclic nitrogen in the 1-naphthol compounds (compare compound **11** with **13**) significantly potentiates the inhibition of *Ee*AChE, affording additional capacity for the inhibition of *eq*BuChE. For the other *imidazopyranotacrines*, it is clear that those bearing an ethoxycarbonyl moiety and a methyl group at C3 and C2, respectively, in the 4*H*-pyran core (**1**, **2**) are more potent than those bearing a fused 3,3-dimethylcyclohexan-1-one (**6**, **5**) or an acetyl and a methyl group (**4**) at C3 and C2, respectively, in the 4*H*-pyran core. Note also that *imidazopyranotacrines* **1** and **2**, differing only in the size of the cycloalkyl ring, are almost equipotent *Ee*AChEIs, while **3**, bearing a cycloheptane ring, is two orders of magnitude less active. Finally, regarding *eq*BuChE inhibition, *imidazopyranotacrines* **1**–**13** were very weak BuChEIs. Because of the weak inhibitory activity, high concentrations were required for the determination of the IC_50_ values, exceeding the solubility of some compounds. Thus, *imidazopyranotacrines* **1**–**13** were screened at a single concentration, namely 10 µM, giving percentages of inhibition between 12.9 and 75.7 (Table 1).

**Figure 3:**
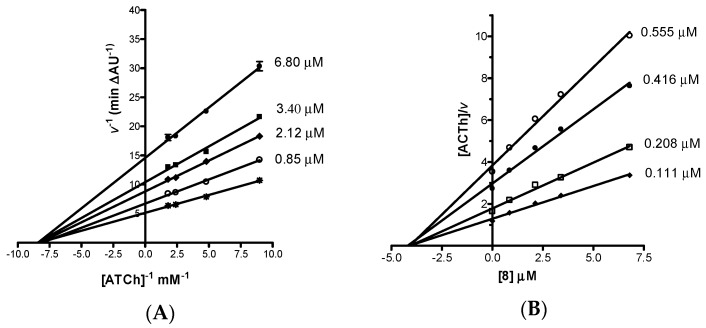
(**A**) Lineweaver-Burk plot and (**B**) Cornish-Bowden plot both illustrating non-competitive inhibition of *Ee*AChE by compound **8**. The intersection point in Cornish-Bowden plot gives an estimate of the inhibition constant. Lines were derived from a weighted least-squares analysis of data. ATCh = acetylthiocholine (substrate).

The mechanism of inhibition of *Ee*AChE by the most potent AChEI, *imidazopyranotacrine* **8**, was investigated by building Lineweaver-Burk double reciprocal plots at increasing concentrations of inhibitor and substrate (acetylthiocholine). Analysis of the Lineweaver-Burk reciprocal plots (Figure 3A) showed that K_m_ appeared unaltered while V_max_ decreased at increasing concentrations of the inhibitor. This pattern indicated that **8** acts as a non-competitive enzyme inhibitor. The non-competitive mechanism of action was confirmed by building the Cornish-Bowden plot (Figure 3B). Replots of the substrate/velocity *versus* the concentration of compound **8** provided an estimate of the non-competitive inhibition constant [29], which resulted equal to 4.10 μM. The non-competitive mechanism of action was confirmed by computational chemistry by performing the docking analysis of both enantiomers of compound **8** on AChE (see Supplementary Materials).

#### 2.2.2. Antioxidant Power

The antioxidant power of the *imidazopyranotacrines* **1**–**13** was determined by the oxygen radical absorbance capacity by fluorescence (ORAC-FL) method [30] using fluorescein as fluorescent probe and Trolox (6-hydroxy-2,5,7,8-tetramethylchroman-2-carboxylic acid) as a reference compound. Results are expressed as Trolox equivalents (μmol Trolox per μmol test compound). As shown in Table 1, all the *imidazopyranotacrines* were able to reduce the amount of peroxyl radical, with ORAC values ranging from 1.35 (**9**) to 2.75 (**10**). The most potent antioxidant agent was **10** (2.75), followed by **6** (2.34), **11** (2.33), **4** (2.31) and **13** (2.25). Although it was clear that the naphthol derivatives, and particularly the 1-naphthol derivatives **10** and **13**, were very potent, we observed that compounds **6** and **4**, bearing a fused 3,3-dimethylcyclohexan-1-one, or an acetyl and a methyl groups at C3 and C2, respectively, in the 4*H*-pyran core, also showed high antioxidant power. Note also that tacrine has a very low ORAC value (0.20 ± 0.04) [28] compared with most of our synthetic *imidazopyranotacrines.*

Next, and on the basis of the AChE inhibition and ORAC values, *imidazopyranotacrines* **2**, **4**, **8** and **10** were evaluated for their potential hepatotoxicity, since tacrine was withdrawn from the market because of its liver toxicity.

#### 2.2.3. Hepatotoxicity Analysis on HepG2 Cells

Drug-induced hepatotoxicity is one of the major reasons for drug [31] withdrawal from the clinic [15], as it happened to tacrine. HepG2 cell is a well-known *in vitro* system to screen the hepatotoxicity activity [32]. As shown in Table 2, tacrine was safe up to 100 µM, but significantly decreased cell viability at 300 μM and at higher concentrations. Among the selected derivatives, compounds **10** and **8** were more toxic than tacrine (significant reduction of cell viability at 30 and 100 µM), *imidazopyranotacrines* **2** and **4** resulted significantly less hepatotoxic than tacrine, and very interestingly, were safe even at 300 µM. *Imidazopyranotacrine* **4** showed the best safety profile, and proved to be non-toxic even at the highest tested concentration, namely 1000 µM, with a recorded cell viability value not significantly different from the control (not significant by the ANOVA test).

## 3. Materials and Methods

### 3.1. General Information

Melting points were determined on a Kofler apparatus, and are uncorrected. IR spectra were recorded on a Shimadzu FT-IR 8201 PC spectrophotometer (Shimadzu, Kyoto, Japan), and only significant absorption bands are reported. ^1^H-NMR and ^13^C-NMR spectra were recorded on a Bruker Avance DPX250 (Bruker Avance DPX250, Bruker BioSpin, Fällanden, Switzerland) or VARIAN Mercury-300 (Palo Alto, CA, USA) spectrometers at 250 or 300 MHz and at 62 or 75 MHz, respectively in CDCl_3_, DMSO-*d*_6_ or CD_3_CN at 250 or 300 MHz and at 62 or 75 MHz, respectively, using solvent peaks as internal reference. Elemental analyses were performed on a Carlo-Erba CHNS apparatus, at CNQO (Madrid, Spain). TLC were performed on precoated Merck (Kenilworth, NJ, USA) silica gel aluminum sheets 60 F254 and detection by UV light at 254 nm or by charring with either ninhydrin, anisaldehyde or phosphomolybdic-H_2_SO_4_ reagents. Anhydrous solvents were used in all experiments. Flash column chromatography was performed on silica gel 60 (230 mesh). Commercial grade reagents were used as supplied from Acros (Geel, Belgium) or Aldrich (Saint Louis, MO, USA).

### 3.2. General Procedure for the Preparation of 1-Methylimidazole-4H-pyran Derivatives

To a solution of the 2-(1-methyl-1*H*-imidazol-2-yl)methylene)malononitrile (**15**) in ethanol (1 mmol/10 mL), the corresponding 1,3-dicarbonyle (1.1 mmol), and some drops of piperidine were added. The mixture was stirred at room temperature (rt) and the reaction was monitored by TLC. The precipitated solid was isolated by filtration, washed with cold ethanol, and purified by silica gel flash chromatography using AcOEt/Hexane (1:1) as eluent to give pure compounds.

### 3.3. General Procedure for the Preparation of 1-Methylimidazole-4H-pyran Derivatives from 1- or 2-Naphthol

A mixture of 1 mmol of 2-((1-methyl-1*H*-(benz)imidazol-2-yl) methylene)malononitrile and 1- or 2-naphthol (1 mmol) in ethanol (10 mL) containing 2–3 drops of piperidine was heated under reflux for overnight. The reaction mixture was cooled and the precipitated product filtered, washed with cold ethanol and purified by silica gel flash chromatography using AcOEt or acetone as eluent to give pure compounds.

### 3.4. General Method for the Friedländer Reaction

Aluminum chloride (1.5 equiv) was suspended in dry 1,2-dichloroethane at rt. Then, the 4*H*-pyran (1 equiv) and the selected cycloalcanone (1.5 equiv) were added, and the reaction mixture was heated at reflux for 24 h. When the reaction was over (thin layer chromatography analysis), an aqueous solution of sodium hydroxide (10%) was added dropwise to the mixture until the aqueous solution became basic. After stirring for 30 min, the precipitate was filtered off and washed with water. The resultant solid was purified by silica gel flash chromatography using AcOEt as eluent to give pure compounds.

*2-(1-Methyl-1H-imidazol-2yl)methylene)malononitrile* (**15**). To a solution of 1-methyl-1*H*-imidazole-2-carbaldehyde (**14**) in ethanol (1.1 g, 10 mmol), 1 equiv (1.58 g, 10 mmol) of malononitrile and 2–3 drops of piperidine were added, and the mixture was stirred at rt overnight. The precipitated product was filtered off and washed with cold ethanol to afford 1.47 g (9.3 mmol) of 2-(1-methyl-1*H*-imidazol-2-yl) methylene)malononitrile (**15**) (93% yield): mp 200–202 °C; IR (KBr) _max_ 2210 (CN) cm^−1^; ^1^H-NMR (250 MHz, DMSO-*d*_6_) δ 8.15 (s, 1H), 7.58 (s, 1H), 7.40 (s, 1H), 3.79 (s, 3H); ^13^C-NMR (62.9 MHz, CD_3_CN) δ 142.7, 141.1, 134.0, 129.2, 115.8, 114.1, 79.3, 34.1. Anal. Calcd. for C_8_H_6_N_4_: C, 60.75; H, 3.82; N, 35.42. Found: C, 60.50; H, 4.03; N, 35.61.

*Ethyl 6-amino-5-cyano-2-methyl-4-(1-methyl-1H-imidazol-2-yl)-4H-pyran-3-carboxylate* (**16**). According to the general procedure, 2-(1-methyl-1*H*-imidazol-2-yl)methylene)malononitrile (**15**) (160 mg, 1.01 mmol), ethyl acetoacetate (150 mg, 1.15 mmol), and piperidine (2-3 drops), gave 270 mg (0.93 mmol) of the title compound **16** (93% yield): mp 230 °C; IR (KBr) _max_ 3390 (NH_2_), 2318 (CN), 1689 (CO) cm^−1^; ^1^H-NMR (300 MHz, DMSO-*d*_6_) δ 6.96–6.94 (m, 3H), 6.69 (s, 1H), 4.59 (s, 1H), 3.99–3.87 (m, 2H), 3.65 (s, 3H), 2.30 (s, 3H), 0.97 (t, *J* = 7.1Hz, 3H); ^13^C-NMR (74.5 MHz, DMSO-*d*_6_) δ 165.2, 158.7, 157.6, 149.6, 126.6, 120.7, 119.8, 105.0, 60.0, 54.5, 32.1, 30.1, 18.2, 13.8. Anal. Calcd. for C_14_H_16_N_4_O_3_: C, 58.32; H, 5.59; N, 19.43. Found: C, 58.09; H, 5.49; N, 19.32.

*5 -Acetyl-2-amino-6-methyl-4-(1-methyl-1H-imidazol-2-yl)-4H-pyran-3-carbonitrile* (**17**). In the same manner, 2-(1-methyl-1*H*-imidazol-2-yl)methylene)malononitrile (**15**) (160 mg, 1.01 mmol), 2,4-pentadione (115 mg, 1.15 mmol), and piperidine (2–3 drops), gave 230 mg (0.89 mmol) of the title compound **17** (90% yield): mp 220 °C; IR (KBr) _max_ 3394 (NH_2_), 2218 (CN), 1616 (CO) cm^−1^; ^1^H-NMR (300 MHz, DMSO-*d*_6_) δ 7.00–6.96 (s, 3H), 6.71 (s, 1H), 4.76 (s, 1H), 3.66 (s, 3H), 2.32 (s, 3H), 2.01 (s, 3H) ; ^13^C-NMR (74.5 MHz, DMSO-*d*_6_) δ 197.5, 159.0, 155.9, 148.8, 126.7, 121.6, 119.9, 113.2, 54.2, 32.3, 30.9, 29.6, 18.8. Anal. Calcd. for C_13_H_14_N_4_O_2_: C, 60.45; H, 5.46; N, 21.69. Found: C, 60.23; H, 5.42; N, 21.55.

*2-Amino-7,7-dimethyl-4-(1-methyl-1H-imidazol-2-yl)-5-oxo-5,6,7,8-tetrahydro-4H-chromene-3-carbonitrile* (**18**). 2-(1-Methyl-1*H*-imidazol-2-yl)methylene)malononitrile (**15**) (160 mg, 1.01 mmol), dimedone (155 mg, 1.1 mmol) and piperidine (2–3 drops), gave 205 mg (0.69 mmol) of the title compound **18** (69% yield): mp 192–193 °C; IR (KBr) _max_ 3367 (NH_2_), 2183 (CN), 1689 (CO) cm^−1^; ^1^H-NMR (300 MHz, DMSO-*d*_6_) δ 7.01 (s, 2H), 6.92 (s, 1H), 6.66 (s, 1H), 4.44 (s, 1H), 3.70 (s, 3H), 2.56–2.03 (m, 4H), 1.03 (s, 3H), 0.99 (s, 3H); ^13^C-NMR (74.5 MHz, DMSO-*d*_6_) δ 196.0, 162.6, 158.9, 149.3, 126.5, 120.6, 119.9, 111.3, 55.5, 49.8, 32.3, 32.1, 25.5, 26.9, 26.5. Anal. Calcd. for C_16_H_18_N_4_O_2_: C, 64.41; H, 6.08; N, 18.78. Found: C, 64.50; H, 6.02; N, 18.94.

*2-Amino-4-(1-methyl-1H-imidazol-2-yl)-4H-benzo[h]chromene-3-carbonitrile* (**20**). According to the general procedure, 2-(1-methyl-1*H*-imidazol-2-yl)methylene)malononitrile (**15**) (160 mg, 1.01 mmol), 1-naphtol (146 mg, 1.01 mmol) and piperidine (2–3 drops), gave 228 mg (0.75 mmol) of the title compound **20** (75% yield): mp > 260 °C; IR (KBr) _max_ 3433 (NH_2_), 2376 (CN) cm^−1^; ^1^H-NMR (250 MHz, CD_3_CN) δ 7.95–7.90 (m, 2H), 7.78–7.75 (m, 1H), 7.53–7.43 (m, 2H), 7.30 (d, *J* = 8.91 Hz, 1H), 7.15 (br,s, 2H), 7.98 (s, 1H), 6.67 (s, 1H), 5.56 (s, 1H), 3.65 (s, 3H); ^13^C-NMR (62.9 MHz, CD_3_CN) δ 160.4, 148.9, 146.8, 130.7, 130.6, 129.8, 128.5, 127.3, 126.6, 125.1, 123.2, 121.9, 120.5, 116.8, 113.1, 53.7, 32.4, 31.5. Anal. Calcd. for C_18_H_14_N_4_O: C, 71.51; H, 4.67; N, 18.53. Found: C, 71.38; H, 4.51; N, 18.46.

*2-Amino-1-(1-methyl-1H-imidazol-2-yl)-1H-benzo[f]chromene-3-carbonitrile* (**19**). Following the same procedure, 2-(1-methyl-1*H*-imidazol-2-yl)methylene)malononitrile (**15**) (160 mg, 1.01 mmol), 2-naphtol (146 mg, 1.01 mmol) and piperidine (2–3 drops), gave 218 mg (0.72 mmol) of the title compound **19** (72% yield): mp > 260 °C; IR (KBr) _max_ 3440 .(NH_2_), 2183 (CN) cm^−1^; ^1^H-NMR (250 MHz, DMSO-*d*_6_) δ 7.96–7.92 (m, 2H), 7.78–7.75 (m, 1H), 7.51–7.43 (m, 2H), 7.30 (d, *J* = 8.9 Hz, 2H), 6.99 (d, *J* = 0.8 Hz, 1H), 6.67 (d, *J* = 0.9 Hz, 1H), 5.67 (s, 1H), 3.66 (s, 3H); ^13^C-NMR (62.9 MHz, DMSO-*d*_6_) δ 160.4, 148.9, 146.8, 130.7, 130.4, 129.7, 128.5, 127.3, 126.6, 125.0, 123.2, 121.9, 120.5, 116.8, 113.1, 53.7, 32.4, 31.4. Anal. Calcd. for C_18_H_14_N_4_O: C, 71.51; H, 4.67; N, 18.53. Found: C, 71.47; H, 4.50; N, 18.72.

*2-Amino-4-(1-methyl-1H-imidazol-2-yl)-4H-pyrano[3,2-h]quinoline-3-carbonitrile* (**21**). Using the procedure described above, 2-(1-methyl-1*H*-imidazol-2-yl) methylene)malononitrile (**15**) (160 mg, 1.01 mmol), 8-hydroxyquinoline (147 mg, 1.01 mmol) and piperidine (2–3 drops), gave 142 mg (0.47 mmol) of the title compound **21** (47% yield): mp 244–246 °C; IR (KBr) _max_ 3398 (NH_2_), 2191 (CN) cm^−1^; ^1^H-NMR (250 MHz, CDCl_3_) δ 8.93–8.91 (m, 1H), 8.22 (dd, *J* = 8.3 Hz, *J* = 1.6 Hz, 1H), 7.59–7.51 (m, 2H), 7.12 (d, *J* = 8.5 Hz, 1H), 6.90–6.84 (m, 4H), 5.83 (s, 1H), 3.56 (s, 3H); ^13^C-NMR (62.9 MHz, CDCl_3_) δ 159.4, 148.9, 146.7, 142.3, 136.5, 134.9, 127.1, 125.7, 124.9, 122.6, 121.3, 120.9, 115.8, 117.1, 52.0, 33.9, 31.7. Anal. Calcd. for C_17_H_13_N_5_O: C, 67.32; H, 4.32 ; N, 23.09. Found: C, 67.55; H, 4.11; N, 23.25.

*Ethyl 5-amino-2-methyl-4-(1-methyl-1H-imidazol-2-yl)-4,6,7,8-tetrahydrocyclopenta[b]pyrano[3,2-e]pyridine-3-carboxylate* (**1**). According to the procedure described above, ethyl 6-amino-5-cyano-2-methyl-4-(1-methyl-1*H*-imidazol-2-yl)-4*H*-pyran-3-carboxylate (**16**) (290 mg, 1.0 mmol), AlCl_3_ (200 mg, 1.53 mmol), and cyclopentanone (130 mg, 1.54 mmol), gave 191 mg (0.54 mmol) of the title compound **1** (54% yield): mp 160–161 °C; IR (KBr) _max_ 3344 (NH_2_); 1704 (CO) cm^−1^; ^1^H-NMR (250 MHz, CDCl_3_) δ 6.85 (s, 1H), 6.66 (s, 1H,), 5.43 (s, 1H^′^), 5.11 (br s, 2H), 4.14–4.02 (m, 2H), 3.42 (s, 3H), 2.87–2.77 (m, 2H), 2.65–2.59 (m, 2H), 2.47 (s, 3H), 2.05–2.02 (m, 2H), 1.14–2.05 (m, 3H); ^13^C-NMR (62.9 MHz, CDCl_3_) δ 166.4, 162.4, 161.3, 155.6, 150.1, 147.0, 126.2, 122.6, 118.1, 101.6, 94.5, 60.4, 34.4, 34.3, 32.9, 27.0, 22.1, 19.3, 14.1. Anal. Calcd. for C_19_H_22_N_4_O_3_: C, 64.39; H, 6.26; N, 15.81. Found: C, 64.16; H, 6.37; N, 15.77.

*Ethyl 5-amino-2-methyl-4-(1-methyl-1H-imidazol-2-yl)-6,7,8,9-tetrahydro-4H-pyrano[2,3-b]quinoline-3-carboxylate* (**2**). Ethyl 6-amino-5-cyano-2-methyl-4-(1-methyl-1*H*-imidazol-2-yl)-4*H*-pyran-3-carboxylate (**16**) (300 mg, 1.04 mmol), cyclohexanone (150 mg, 1.53 equiv), and AlCl_3_ (200 mg, 1.53 mmol), gave 272 mg (0.74 mmol) of the title compound **2** (72% yield): mp 222–224 °C; IR (KBr) _max_ 3343 (NH_2_); 1701 (CO) cm^−1^; ^1^H-NMR (250 MHz, CDCl_3_) δ 6.86 (s, 1H), 6.67 (s, 1H), 5.42 (s, 1H), 5.15 (br s, 2H), 4.22–3.99 (m, 2H), 3.43 (s, 3H), 2.70–2.68 (m, 2H), 2.49 (s, 3H), 2.35–2.17 (m, 2H), 1.73–1.15 (m, 4H), 1.13–1.10 (m, 3H); ^13^C-NMR (62.9 MHz, CDCl_3_) δ 166.5, 161.4, 154.3, 153.4, 152.1, 147.0, 126.2, 122.6, 113.6, 101.7, 94.3, 60.5, 34.5, 33.0, 32.5, 22.8, 22.5, 22.3, 19.4, 14.2. Anal. Calcd. for C_20_H_24_N_4_O_3_: C, 65.20; H, 6.57; N, 15.21. Found: C, 65.41; H, 6.33; N, 15.02.

*Ethyl 5-amino-2-methyl-4-(1-methyl-1H-imidazol-2-yl)-4,6,7,8,9,10-hexahydrocyclohepta[b]pyrano[3,2-e]pyridine-3-carboxylate* (**3**). Following the general procedure, ethyl 6-amino-5-cyano-2-methyl-4-(1-methyl-1*H*-imidazol-2-yl)-4*H*-pyran-3-carboxylate (**16**) (300 mg, 1.04 mmol), AlCl_3_ (200 mg, 1.53 mmol), cycloheptanone (170 mg, 1.51 mmol), gave 295 mg (0.77 mmol) of the title compound **3** (74% yield): mp 196–198 °C; IR (KBr) _max_ 3343 (NH_2_); 1701 (CO) cm^−1^; ^1^H-NMR (250 MHz, CDCl_3_) δ 6.82 (s, 1H), 6.65 (s, 1H), 5.35 (s, 1H), 5.19 (br s, 2H), 4.16–4.01 (m, 2H), 3.40 (s, 3H), 2.81–2.77 (m, 2H,), 2.45–2.40 (m, 5H), 1.79–1.42 (m, 6H,), 1.12–1.14 (m, 3H); ^13^C-NMR (62.9 MHz, CDCl_3_) δ 166.3, 161.2, 160.8, 152.9, 151.1, 146.8, 126.1, 122.6, 118.8, 101.7, 95.1, 60.3, 38.5, 34.5, 32.9, 32.0, 29.6, 26.6, 26.0, 25.5, 19.2, 14.1. Anal. Calcd. for C_21_H_26_N_4_O_3_: C, 65.95; H, 6.85; N, 14.65. Found: C, 66.01; H, 6.66; N, 14.84.

*1-(5-Amino-2-methyl-4-(1-methyl-1H-imidazol-2-yl)-6,7,8,9-tetrahydro-4H-pyrano[2,3-b]quinolin-3-yl)ethan-1-one* (**4**). In accordance with the general procedure, 5-acetyl-2-amino-6-methyl-4-(1-methyl-1*H*-imidazol-2-yl)-4*H*-pyran-3-carbonitrile (**17**) (260 mg, 1.0 mmol), cyclohexanone (150 mg, 1.53 mmol), and AlCl_3_ (200 mg, 1.53 mmol), gave 175 mg (0.52 mmol) of the title compound **4** (52% yield): mp 243–245 °C; IR (KBr) _max_ 3359 (NH_2_); 1689 (CO) cm^−1^; ^1^H-NMR (250 MHz, CDCl_3_): δ 6.84 (s, 1H), 6.68 (s, 1H), 5.39 (s, 1H), 5.07 (br s, 2H), 3.37 (s, 3H), 2.65–2.49 (m, 2H), 2.36 (s, 3H), 2.23–2.15 (m, 5H), 1.75–1.67 (m, 4H); ^13^C-NMR (62.9 MHz, CDCl_3_) δ 199.1, 159.2, 154.5, 153.2, 151.9, 146.2, 126.3, 123.3, 113.5, 109.4, 93.9, 35.2, 33.0, 32.4, 29.7, 22.7, 22.4, 22.2, 19.6. Anal. Calcd. for C_19_H_22_N_4_O_2_: C, 67.44; H, 6.55; N, 16.56. Found: C, 67.31; H, 6.63; N, 16.44.

*11-Amino-7,7-dimethyl-10-(1-methyl-1H-imidazol-2-yl)-2,3,6,7,8,10-hexahydrochromeno[2,3-b]cyclopenta[e]pyridin-9(1H)-one* (**5**). According to the procedure, 2-amino-7,7-dimethyl-4-(1-methyl-1*H*-imidazol-2-yl)-5-oxo-5,6,7,8-tetrahydro-4*H*-chromene-3-carbonitrile (**18**) (300 mg, 1.0 mmol), anhydrous AlCl_3_ (200 mg, 1.5 mmol) in dry ClCH_2_CH_2_Cl (10 mL), and cyclopentanone (130 mg, 1.54 mmol) gave 185 mg (0.51 mmol) of the title compound **5** (51% yield): mp > 260 °C; IR (KBr) _max_ 3442 (NH_2_), 1643 (CO) cm^−1^; ^1^H-NMR (250 MHz, CDCl_3_) δ 6.80 (s, 1H), 6.67 (s, 1H), 5.29 (s, 1H), 4.98 (br s, 2H), 3.41 (s, 3H), 2.60–2.57 (m, 2H), 2.43–2.22 (m, 4H), 2.15–2.06 (m, 2H), 1.96–1.04 (m, 2H), 0.99 (s, 6H); ^13^C-NMR (62.9 MHz, CDCl_3_) δ 196.0, 165.7, 162.0, 155.4, 150.2, 146.7, 126.6, 122.0, 118.4, 109.0, 95.4, 50.5, 41.2, 34.0, 32.7, 31.8, 30.3, 28.3, 28.2, 26.9, 22.0. Anal. Calcd. for C_21_H_24_N_4_O_2_: C, 69.21; H, 6.64; N, 15.37. Found: C, 69.01; H, 6.44; N, 15.55.

*11-Amino-3,3-dimethyl-12-(1-methyl-1H-imidazol-2-yl)-2,3,4,7,8,9,10,12-octahydro-1H-chromeno[2,3-b]quinolin-1-one* (**6**). Using the same procedure, 2-amino-7,7-dimethyl-4-(1-methyl-1*H*-imidazol-2-yl)-5-oxo-5,6,7,8-tetrahydro-4*H*-chromene-3-carbonitrile (**18**) (300 mg, 1.0 mmol), AlCl_3_ (200 mg, 1.53 mmol), and cyclohexanone (150 mg, 1.53 mmol), gave 132 mg (0.35 mmol) of the title compound **6** (35% yield): mp > 260 °C; IR (KBr) _max_ 3341 (NH_2_); 1702 (CO) cm^−1^; ^1^H-NMR (250 MHz, CDCl_3_) δ 6.94 (s, 1H), 6.69 (s, 1H), 5.36 (s, 1H), 4.99 (br s, 2H), 3.49 (s, 3H), 2.75–2.61 (m, 4H), 2.40–2.20 (m, 4H), 1.83–1.31 (m, 4H), 1.14 (s, 6H); ^13^C-NMR (62.9 MHz, CDCl_3_) δ 196.3, 166.0, 161.2, 154.4, 152.4, 146.9, 127.1, 122,3, 114,1 109.4, 95.4, 50.8, 41.6, 33.0, 32.5, 32.2, 30.8, 28.6, 22.9, 22.6, 22.4; Anal. Calcd. for C_22_H_26_N_4_O_2_: C, 69.82; H, 6.92; N, 14.80. Found: C, 69.66; H, 6.78; N, 14.67.

*13-(1-Methyl-1H-imidazol-2-yl)-9,10,11,13-tetrahydrobenzo[5,6]chromeno[2,3-b]cyclopenta[e]pyridin-12-amine* (**7**). In accordance with the general procedure, 2-amino-1-(1-methyl-1*H*-imidazol-2-yl)-1*H*-benzo[*f*]chromene-3-carbonitrile (**19**) (300 mg, 1.0. mmol), AlCl_3_ (200 mg, 1.53 mmol), cyclopentanone (130 mg, 1.54 mmol), gave 202 mg (0.55 mmol) of the title compound **7** (55% yield): mp > 260 °C; IR (KBr) _max_ 3352 (NH_2_) cm^−1^; ^1^H-NMR (250 MHz, CDCl_3_) δ 8.20 (d, *J* = 8.4 Hz, 1H), 7.86–7.82 (m, 2H),7.50–7.39 (m, 3H), 6.96 (s, 1H), 6.59 (s, 1H), 6.29 (s, 1H), 5.32 (br s, 2H), 3.08 (s, 3H), 2.99–2.91 (m, 2H), 2.77–2.64 (m, 2H), 2.21–2.09 (m, 2H); ^13^C-NMR (62.9 MHz, CDCl_3_) δ 162.8, 156.6, 150.6, 149.5, 147.8, 131.7, 130.8, 130.3, 128.5, 127.5, 126.3, 124.7, 123.5, 123.1, 118.0, 117.6, 110.5, 94.7, 34.8, 34.4, 32.9, 27.3, 22.1. Anal. Calcd. for C_23_H_20_N_4_O: C, 74.98; H, 5.47; N, 15.21. Found: C, 75.01; H, 5.51; N, 15.02.

*14-(1-Methyl-1H-imidazol-2-yl)-9,11,12,14-tetrahydro-10H-benzo[5,6]chromeno[2,3-b]quinolin-13-amine* (**8**). Following the general procedure, 2-amino-1-(1-methyl-1*H*-imidazol-2-yl)-1*H*-benzo[*f*]chromene-3-carbonitrile (**19**) (300 mg, 1.0 mmol), AlCl_3_ (200 mg, 1.53 mmol), and cyclohexanone (150 mg, 1.53 mmol), gave 225 mg (0.59 mmol) of the title compound **8** (59% yield): mp > 260 °C; IR (KBr) _max_ 3351 (NH_2_) cm^−1^; ^1^H-NMR (250 MHz, CDCl_3_) δ 8.20 (d, *J* = 8.3 Hz, 1H), 7.84–7.80 (m, 2H), 7.52–7.37 (m, 3H), 6.94 (s, 1H), 6.56 (s, 1H), 6.25 (s, 1H), 5.37 (br s, 2H), 3.08 (s, 3H), 2.88–2.72 (m, 2H), 2.44–2.26 (m, 2H), 1.85–1.77 (m, 4H); ^13^C-NMR (62.9 MHz, CDCl_3_) δ 154.5, 154.4, 152.6, 149.5, 147.6, 131.7, 130.7, 130.3, 128.5, 127.4, 126.1, 124.6, 123.4, 122.9, 118.0, 112.9, 110.5, 94.4, 34.7, 32.8, 32.6, 22.9, 22.7, 22.5. Anal. Calcd. for C_24_H_22_N_4_O: C, 75.37; H, 5.80; N, 14.65. Found: C, 75.30; H, 5.82; N, 14.38.

*15-(1-Methyl-1H-imidazol-2-yl)-9,10,11,12,13,15-hexahydrobenzo[5,6]chromeno[2,3-b]cyclohepta[e]pyridin-14-amine* (**9**). Following the general procedure, 2-amino-1-(1-methyl-1*H*-imidazol-2-yl)-1*H*-benzo[*f*]chromene-3-carbonitrile (**19**) 300 mg (1.0. mmol), AlCl_3_ (200 mg, 1.53 mmol), and cycloheptanone (170 mg, 1.51 mmol), gave 238 mg (0.6 mmol) of the title compound **9** (60% yield): mp > 260 °C; IR (KBr) _max_ 3355 (NH_2_ cm^−1^); ^1^H-NMR (250 MHz, CDCl_3_) δ 8.21 (d, *J* = 8.2 Hz, 1H), 7.84–7.80 (m, 2H), 7.52–7.37 (m, 3H), 6.95 (s, 1H), 6.57 (s, 1H), 6.32 (s, 1H), 5.43 (br s, 2H), 3.09 (s, 3H), 2.96–2.92 (m, 2H), 2.57–2.53 (m, 2H), 1.87–1.53 (m, 6H); ^13^C-NMR (62.9 MHz, CDCl_3_) δ 161.1, 154.1, 151.7, 149.5, 147.6, 131.7, 130.7, 130.3, 128.5, 127.4, 126.2, 124.6, 123.4, 123.0, 118.3, 117.9, 110.6, 95.4, 38.9, 35.0, 32.8, 32.3, 26.9, 26.2, 25.7. Anal. Calcd. For C_25_H_24_N_4_O: C, 75.73; H, 6.10; N, 14.13. Found: C, 75.66; H, 6.23; N, 14.01.

*7-(1-Methyl-1H-imidazol-2-yl)-9,10,11,12-tetrahydro-7H-benzo[7,8]chromeno[2,3-b]quinolin-8-amine* (**10**). Following the general procedure, 2-amino-4-(1-methyl-1*H*-imidazol-2-yl)-4*H*-benzo[*h*]chromene-3-carbonitrile (**20**) (300 mg, 1.0 mmol), cyclohexanone (150 mg, 1.53 mmol), and AlCl_3_ (200 mg, 1.53 mmol), gave 198 mg (0.52 mmol) of the title compound **10** (52% yield): mp > 260 °C; IR (KBr) _max_ 3355 (NH_2_) cm^−1^; ^1^H-NMR (250 MHz, CDCl_3_) δ 8.27 (d, *J* = 8.3 Hz, 1H), 7.85–7.81 (m, 2H),7.50–7.40 (m, 3H), 6.95 (s, 1H), 6.58 (s, 1H), 6.26 (s, 1H), 5.32 (br s, 2H), 3.09 (s, 3H), 2.81–2.80 (m, 2H), 2.45–2.25 (m, 2H), 1.86–1.78 (m, 4H); ^13^C-NMR (62.9 MHz, CDCl_3_) δ 154.6, 154.5, 152.6, 149.5, 147.7, 131.7, 130.7, 130.3, 128.5, 127.5, 126.1, 124.7, 123.5, 122.9, 118.0, 113.0, 110.6, 94.5, 34.8, 32.8, 32.7, 22.9, 22.7, 22.5. Anal. Calcd. for C_24_H_22_N_4_O: C, 75.37; H, 5.80; N, 14.65. Found: C, 75.12; H, 5.75; N, 14.58.

*7-(1-Methyl-1H-imidazol-2-yl)-7,9,10,11,12,13-hexahydrobenzo[7,8]chromeno[2,3-b]cyclohepta[e]pyridin-8-amine* (**11**). Following the general procedure, 2-amino-4-(1-methyl-1*H*-imidazol-2-yl)-4*H*-benzo[*h*]chromene-3-carbonitrile (**20**) (300 mg, 1.0 mmol), cycloheptanone (170 mg, 1.51 mmol), and AlCl_3_ (200 mg, 1.53 mmol), gave 245 mg (0.62 mmol) of the title compound **11** (62% yield): mp > 260 °C; IR (KBr) _max_ 3350 (NH_2_) cm^−1^; ^1^H-NMR (250 MHz, CDCl_3_) δ 8.21 (d, *J* = 8.3 Hz, 1H), 7.84–7.80 (m, 2H), 7.51–7.42 (m, 3H), 6.99 (s, 1H), 6.58 (s, 1H), 6.25 (s, 1H), 5.43 (br s, 2H), 3.00 (s, 3H), 2.96–2.92 (m, 2H), 2.57–2.52 (m, 2H), 1.88–1.27 (m, 6H); ^13^C-NMR (62.9 MHz, CDCl_3_) δ 161.1, 154.1, 151.7, 149.5, 147.6, 131.6, 130.7, 130.3, 128.5, 127.4, 126.1, 124.7, 123.4, 123.0, 118.3, 117.9, 110.6, 95.3, 38.9, 34.9, 32.8, 32.3, 26.9, 26.5, 25.7. Anal. Calcd. for C_25_H_24_N_4_O: C, 75.73; H, 6.10; N, 14.13. Found: C, 75.88; H, 6.02; N, 14.25.

*7-(1-Methyl-1H-imidazol-2-yl)-7,9,10,11-tetrahydrocyclopenta[5′,6′]pyrido[3′,2′:5,6]pyrano[3,2-h]quinolin-8-amine* (**12**). In accordance with the general procedure, 2-amino-4-(1-methyl-1*H*-imidazol-2-yl)-4*H*-pyrano[3,2-*h*]quinoline-3-carbonitrile (**21**) (1.0 mmol, 300 mg), AlCl_3_ (200 mg, 1.5 mmol) and cyclopentanone (130 mg, 1.54 mmol), gave 238 mg (0.65 mmol) of title compound **12** (65% yield): mp > 260 °C; IR (KBr) _max_ 3349 (NH_2_) cm^−1^; ^1^H-NMR (250 MHz, CDCl_3_) δ 9.00–8.98 (m, 1H), 8.11–8.07 (m, 1H), 7.47–7.44 (m, 2H), 7.34–7.23 (m, 1H), 6.94 (s, 1H), 6.71 (s, 1H), 5.89 (s, 1H), 4.92 (br s, 2H), 3.10 (s, 3H), 2.95–2.87 (m, 2H), 2.70–2.59 (m, 2H), 2.15–2.04 (m, 2H); ^13^C-NMR (62.9 MHz, CDCl_3_) δ 163.1, 156.7, 150.4, 150.2, 147.8, 146.1, 138.5, 135.6, 128.6, 126.4, 126.3, 123.3, 122.5, 122.1, 117.5, 116.3, 93.5, 36.9, 34.3, 33.0, 27.1, 22.2. Anal. Calcd. for C_22_H_19_N_5_O: C, 71.53; H, 5.18; N, 18.96. Found: C, 71.48; H, 5.33; N, 19.01.

*7-(1-Methyl-1H-imidazol-2-yl)-7,9,10,11,12,13-hexahydrocyclohepta[5′,6′]pyrido[3′,2′:5,6]pyrano[3,2-h]quinolin-8-amine* (**13**). In accordance with the general procedure, 2-amino-4-(1-methyl-1*H*-imidazol-2-yl)-4*H*-pyrano[3,2-*h*]quinoline-3-carbonitrile (**21**) (300 mg, 1.0 mmol), AlCl_3_ (200 mg, 1.5 mmol), and cycloheptanone (170 mg, 1.51 mmol), gave 158 mg (0.40 mmol) of title compound **13** (40% yield): mp 206–207 °C; IR (KBr) _max_ 3429 (NH_2_) cm^−1^; ^1^H-NMR (250 MHz, CDCl_3_) δ 9.00–8.98 (m, 1H), 8.06–8.03 (m, 1H), 7.43–7.36 (m, 2H), 7.28–7.21 (m, 1H), 6.89 (s, 1H), 6.65 (s, 1H), 5.77 (s, 1H), 4.99 (br s, 2H), 3.04 (s, 3H), 2.90–2.76 (m, 2H), 2.63–2.47 (m, 2H), 1.77–1.46 (m, 6H); ^13^C-NMR (62.9 MHz, CDCl_3_) δ 161.5, 153.9, 151.1, 150.3, 147.6, 145.9, 138.3, 135.5, 128.4, 126.4, 126.3, 123.2, 122.3, 122.0, 118.1, 116.4, 94.2, 38.4, 37.1, 32.8, 32.1, 26.8, 26.1, 25.5. Anal. Calcd. for C_24_H_23_N_5_O: C, 72.52; H, 5.83; N, 17.62. Found: C, 72.67; H, 5.85; N, 17.41.

### 3.5. In Vitro Toxicity in HepG2 Cells

#### 3.5.1. Cell Culture and Treatment

The human hepatoma HepG2 line cells were purchased from American Type Culture Collection. The cells were cultured in Eagle’s Minimum Essential Medium (Ozyme, Montigny-le-Bretonneux, France) supplemented with 10% fetal bovine serum, 1X non-essential amino acids, 100 units/mL penicillin and 10 mg/mL streptomycin (Dutscher, Brumath, France). Cultures were kept under a CO_2_/air (5%/95%) humidified atmosphere at 37 °C. Prior to the experiment, cells were seeded in 96-well culture plates at a density of 0.1 × 10^6^ cells per well. After 24 h of incubation, the culture medium was refreshed and 100 µL of the test compounds or DMSO (0.1%) were added. Compounds were tested at 7 concentrations (1–1000 µM) in triplicate.

#### 3.5.2. Measurement of Cell Viability

Cell viability, measured as the mitochondrial activity of living cells, was determined by quantitative colorimetric assay with 3-[4,5 dimethylthiazol-2-yl]-2,5-diphenyl-tetrazolium bromide (MTT) [33]. Briefly, after 24 h of treatment, cells were incubated with 50 µL MTT (0.5 mg/mL, Sigma Aldrich) at 37 °C for 2 h. Plates were centrifuged, MTT was removed and 100 µL DMSO was distributed per well. The absorbance was read at 570 nm by microplate spectrophotometry. Cell viability was expressed as percentage over controls (DMSO, 0.1%).

### 3.6. Measurement of the Inhibitory Potency against EeAChE

The inhibitory activity on AChE from *Electrophorus electricus* (type VI-S, Sigma, Milan, Italy), was evaluated spectrophotometrically by the method of Ellman *et al.* [27]. AChE stock solution was prepared by dissolving *Ee*AChE lyophilized powder (Sigma) in 0.1 M phosphate buffer (pH = 8.0) containing 0.1% Triton X-100. Stock solutions of the tested compounds (1 mM) were prepared in MeOH/H_2_O 50/50 (*v*/*v*) and diluted in MeOH/H_2_O 50/50 (*v*/*v*). Five increasing concentrations of each inhibitor were used, able to give an inhibition of the enzymatic activity in the range of 20–80%. The assay solution consisted of a 0.1 M phosphate buffer pH 8.0, with the addition of 340 μM 5,5′-dithio-bis(2-nitrobenzoic acid), 0.02 unit/mL of *Ee*AChE and 550 μM of substrate (acetylthiocholine iodide, ATCh). Assay solutions with and without inhibitor were preincubated at 37 °C for 20 min followed by the addition of substrate. Blank solutions containing all components except *Ee*AChE were prepared in parallel to account for the non-enzymatic hydrolysis of the substrate. Initial rate assays were performed at 37 °C with a Jasco V-530 double beam spectrophotometer. The rate of increase in the absorbance at 412 nm was followed for 210 s. The velocity in the presence and absence of inhibitor were compared and % inhibition was calculated. The results were plotted by placing the percentage of inhibition in function of the decimal log of the final inhibitor concentration. Linear regression and IC_50_ values were calculated using Microcal Origin 3.5 software (Microcal Software, Kiev, Ukraine).

### 3.7. Measurement of the Inhibitory Potency against eqBuChE

The % inhibitory activity on BuChE from horse serum (lyophilized powder, Sigma-Aldrich), was evaluated spectrophotometrically by the method of Ellman *et al.* [27]. BuChE stock solutions were first dissolved in 0.1 M phosphate buffer (pH 8.0) and then aliquoted in small vials. Compounds were dissolved in DMSO at 10 mM. The reaction was carried out in a final volume of 3 mL of a 0.1 M phosphate-buffered solution at pH 8.0, containing 5,5′-dithiobis-2-nitrobenzoic acid (DTNB, 2625 µL, 0.35 mM, final concentration), *eq*BuChE (60 µL, 0.05 U/mL final concentration), tested compound (3 µL, 10 µM, final concentration) and 1% *w/v* Bovine Serum Albumin phosphate-buffered (pH 8.0) solution (BSA, 40 µL). The inhibition with respect to control without compound was determined by pre-incubating this mixture at rt with each compound for 10 min. After this period, butyrylthiocholine iodide (150 µL, 0.5 mM final concentration) was added and incubated for 15 min at rt. Then the absorbances were measured at 412 nm in a spectrophotometer plate reader (iEMS Reader MF, Labsystems). % inhibition given by each compound was calculated and data are expressed as mean ± SEM of at least three different experiments.

### 3.8. Kinetic Analysis of the Inhibition of EeAChE by Compound ***8***

To obtain estimates of the inhibition mechanism, reciprocal plots of 1/*v vs.* 1/[S] were constructed at different concentrations of the substrate acetylthiocholine (0.111–0.557 mM) by using Ellman′s method [27] and *Ee*AChE (type VI-S, Sigma). Four concentrations of inhibitor were selected for this study: 0.850, 2.124, 3.399, 6.798 μM. The plots were assessed by a weighted least-squares analysis that assumed the variance of the velocity (*v*) to be a constant percentage of *v* for the entire data set. Data analysis was performed with GraphPad Prism 4.03 software (GraphPad Software Inc., San Diego, CA, USA). The determination of the non-competitive inhibition constant *K_i_* value was carried out using Dixon plot, *i.e.*, plotting the apparent 1/*v vs.* inhibitor concentration [I] [34] and confirmed by Cornish-Bowden plot ([substrate]/*v vs.* [I]) [28].

### 3.9. Oxygen Radical Absorbance Capacity Assay

The radical scavenging activity of *imidazopyranotacrines* **1**–**13** was determined by the ORAC-FL method [29,30] using a Varioskan Flash plate reader with built-in injectors (Thermo Scientific). Trolox, 2,2′-azobis(amidinopropane) dihydrochloride (AAPH) and fluorescein (FL) were purchased from Sigma-Aldrich. The reaction was performed at 37 °C in 75 mM phosphate buffer (pH = 7.4), and the final volume reaction mixture was 200 µL. The final concentrations were 1–8 µM for Trolox and 0.1–1 µM for the tested compounds. Briefly, in a black 96-well microplate (Nunc), antioxidant (20 µL) and fluorescein (120 µL) were incubated for 15 min at 37 °C. Then, 2,2′-azobis(amidinopropane) dihydrochloride (60 µL) was added using the built-in injector. The fluorescence at λem 535 nm (λexc = 485 nm) was measured each minute during 60 min. All assays were made in triplicate and at least three different assays were performed for each sample. Antioxidant curves were first normalized to the curve of the blank and then, the area under the fluorescence decay curve (AUC) was calculated as:

AUC = 1 + sum(*fi*/*f*_0_) (1)

where *f*_0_ is the initial fluorescence reading at 0 min and *fi* is the fluorescence value at time *i*.

The net AUC corresponding to a sample was calculated as follows:

Net AUC = AUC_antioxidant_ − AUC_blank_ (2)

Regression equations were calculated by plotting the net AUC against the antioxidant concentration. The ORAC value was obtained by dividing the slope of the latter curve by the slope of the Trolox curve obtained in the same assay. ORAC values was expressed as µmol of Trolox equivalent/µmol of *imidazopyranotacrines*. Data are expressed as mean ± SD.

## 4. Conclusions

Although the etiology of AD is a matter of debate, a number of biological targets [35] such as inhibition of Aβ oligomerization and deposition in senile plaques, halting neurofibrillary tangle formation, and inhibition of γ- and β-secretase [36] have been identified and exploited to design new therapies. However, the recent failure in clinical phase III of semagacestat [37], γ-secretase inhibitor, as well as the clinical phases discontinuation of tideglusib [38], a GSK-3 inhibitor targeting tau protein, clearly show that the soundest hypotheses for AD have not yet provided any drug for AD patients cure. Thus, this may be the moment to repurpose old strategies such as the search for non-hepatotoxic tacrines.

In this context, in this article we have reported our most recent endeavors on this topic [19], describing a number of *imidazopyranotacrines*, a new type of tacrine analogues [39,40,41,42,43], as modest and selective *Ee*AChE inhibitors showing good antioxidant power. Particularly attractive in this family of compounds is the *imidazopyranotacrine* **4**, which is a modest AChEI, but a potent antioxidant agent, endowed with a very interesting safety profile on HepG2 cells, *i.e.*, showing lack of hepatotoxicity even at very high concentration (1000 µM). Other tacrine analogues such as **8** and **10** are significantly more potent AChEIs than **4** and show good antioxidant power, but also, unfortunately, higher hepatotoxicity, although compare favorably with tacrine especially at high concentrations (≥ 300 μM). Kinetic analysis showed that compound **8** is a non-competitive AChEI, a mechanism that we have confirmed by computational chemistry by performing the docking analysis of both enantiomers of compound **8** on AChE. In addition, modeling results showed that the best binding positions for compound **8** on BuChE were clustered rather far from the active site, at the top of the gorge, explaining thus why this compound is a poor *eq*BuChE inhibitor (Supplementary Materials).

We have also calculated the Admission, Distribution, Metabolism, Excretion (ADME) properties of some selected *imidazopyranotacrines*. Thus, about 45 physically significant descriptors and pharmacologically relevant properties of both enantiomers of compounds **2**, **4**, **8** and **10** were predicted and some of the important properties were analyzed. All compounds showed significant values for the properties analyzed and showed drug-like characteristics based on Lipinski′s rule of five (see Table S1, Supplementary Materials). Drugs currently used for neurological disorders treatment are generally CNS acting drugs, which show values of MW, Hydrogen Bond (HB) donor, acceptors and rotatable bonds, *etc.*, in general, in a smaller range than non-CNS therapeutics. Thus, factors that are relevant to the success of CNS drugs were analyzed. CNS drugs show values of MW < 450, HB donor < 3, HB acceptors < 7, QPlogPo/w < 5, PSA < 90, number of rotatable bonds < 8 and hydrogen bonds < 8. Thus, the enantiomers of compounds **2**, **4**, **8** and **10** satisfied all the characteristic of CNS acting drugs. The solubility of organic molecules in water has a significant impact on many ADME-related properties. These compounds present solubility values within the limits. The partition coefficient (QPlogPo/w), critical for estimation of absorption within the body, ranged between 2.82 and 4.75. The percentage human oral absorption for the compounds is high, 100%. The most used parameter for Blood Brain (BB) barrier penetration is log BB. The log BB of many prescribed CNS drugs is >−0.5 and compounds with log BB <−1.0 penetrate poorly into the brain, yet some commercial CNS drugs have log BB <−1.0. For compounds **2**, **4**, **8** and **10**, these values lie within the indicated limits of −3 < QPlogBB < 1.2.

In conclusion, some *imidazopyranotacrine* derivatives showing selective modest AChE inhibition, whose pharmacokinetic parameters are within the acceptable range defined for human use, thereby indicating their potential as drug-like molecules and possible brain penetration, have been discovered for the potential treatment of AD.

## Figures and Tables

**Table 1:** Inhibitory potencies, expressed as IC_50_ values (μM), for the inhibition of *Ee*AChE and *eq*BuChE, and antioxidant power of imidazopyranotacrines **1**–**13**.

Compound	*Ee*AChE ^a^ IC_50_ Values (μM)	*eq*BuChE ^b^ % Inhibition at 10 μM	ORAC ^d^
**1**	12.9 ± 0.3	25.6 ± 0.7	0.34 ± 0.03
**2**	11.5 ± 0.4	34.0 ± 0.5	1.79 ± 0.17
**3**	432 ± 84	32.2 ± 0.9	1.99 ± 0.23
**4**	38.7 ± 1.7	24.9 ± 0.9	2.31 ± 0.29
**5**	158 ± 33	12.9 ± 0.5	1.70 ± 0.33
**6**	37.8 ± 1.2	33.5 ± 1.7	2.34 ± 0.31
**7**	8.41 ± 0.14	50.5 ± 7.3	1.47 ± 0.13
**8**	6.73 ± 0.52	51.6 ± 0.6	1.88 ± 0.01
**9**	na	15.1 ± 4.7	1.35 ± 0.13
**10**	10.7 ± 0.2	25.0 ± 0.2	2.75 ± 0.09
**11**	na	20.3 ± 3.7	2.33 ± 0.13
**12**	15.3 ± 0.6	27.5 ± 1.1	1.88 ± 0.07
**13**	30.0 ± 2.0	75.7 ± 2.3	2.25 ± 0.29
**Tacrine**	0.0898 ± 0.0022	0.005 ± 0.001 ^a,c^	0.20 ± 0.04 [28]

**^a^** Each IC_50_ value is the mean ± SEM of at least two independent experiments each performed in triplicate. **^b^** % inhibition at 10 µM is the mean ± SEM of at least three independent experiments. **^c^** IC_50_ value of tacrine was given. **^d^** Data expressed as µmol Trolox/µmol compound. na: not significantly active (% inhibition lower than 15%) when tested at the highest concentration (solubility limit), namely 15 μM and 25 μM for **9** and **11**, respectively.

**Table 2:** *In vitro* toxicity of *imidazopyranotacrines*, and tacrine in HepG2 cells.

Compounds	1 µM	3 µM	10 µM	30 µM	100 µM	300 µM	1000 µM
**Tacrine**	95.8 ± 2.2	100.4 ± 2.2	96.9 ± 3.9	97.7 ± 3.9	98.6 ± 3.8	39.2 ± 4.7 ***	20.0 ± 2.1 ***
**10**	101.8 ± 4.2	100.7 ± 2.6	100.5 ± 1.9	71.4 ± 7.4 *	63.5 ± 6.2 *	64.8 ± 5.7 *	66.8 ± 4.1 *
**8**	108.1 ± 5.4	101.3 ± 10.8	79.0 ± 9.1	57.6 ± 4.2 **	52.7 ± 3.3 **	51.2 ± 2.8 **	66.2 ± 6.8 *
**2**	102.7 ± 3.7	103.2 ± 4.4	101.3 ± 3.9	99.2 ± 2.6	93.0 ± 4.7	87.5 ± 3.2	58.8 ± 2.1 *
**4**	107.7 ± 5.1	105.3 ± 2.9	102.1 ± 2.9	98.0 ± 3.1	104.6 ± 4.5	97.1 ± 3.0	96.8 ± 1.5

Cell viability was measured as MTT reduction and data were normalized as % control. Data are expressed as the mean ± S.E.M. of triplicate of at least three different cultures. All compounds were assayed at increasing concentrations (1–1000 μM). *** *p* ≤ 0.001, ** *p* ≤ 0.01, * *p* ≤ 0.05, with respect to control group. Comparisons between tested compounds and control group were performed by one-way ANOVA.

## Supplementary Materials

Supplementary materials can be accessed at: http://www.mdpi.com/1420-3049/21/4/400/s1.
