# Supplementary material for: Imidazopyranotacrines as Non-Hepatotoxic, Selective Acetylcholinesterase Inhibitors, and Antioxidant Agents for Alzheimer′s Disease Therapy
Source: Molecules. 2016 Mar 24;21(4):400. doi: 10.3390/molecules21040400 (PMC6273229; doi:10.3390/molecules21040400)
Supplement: Supplementary file 1 [file molecules-21-00400-s001.pdf]

Supplementary Materials: *Imidazopyranotacrines* as Non-hepatotoxic, Selective Acetylcholinesterase Inhibitors, and Antioxidant Agents for Alzheimer's Disease Therapy

Houssem Boulebd, Lhassane Ismaili, Manuela Bartolini, Abdelmalek Bouraiou, Vincenza Andrisano, Helene Martin, Alexandre Bonet, Ignacio Moraleda, Isabel Iriepa, Mourad Chioua, Ali Belfaitah and José Marco-Contelles

Table of Contents

1. Computational chemistry: Molecular modeling of *imidazopyranotacrine* 8..................S2–S5
Molecular modeling methods..S4–S5
2. ADMET of *imidazopyranotacrines* 2, 4, 8 and 10..S5
3. References……………………………………………………………………………..S6

1. Computational Chemistry: Molecular Modeling of *imidazopyranotacrine* 8

A modeling study was carried out through docking simulations to shed light on the nature and spatial location of the key interactions of the (*R*)- and (*S*)-enantiomers of **8**, selected as the most potent ChEI, on the AChE binding, by using AutoDockVina [1].

The kinetic data provide evidence that compound **8** displays a non-competitive type inhibition and argue in favor of interactions of compound **8** with the peripheral anionic site (PAS) of AChE. Molecular modeling studies have been carried in order to validate this assumption.

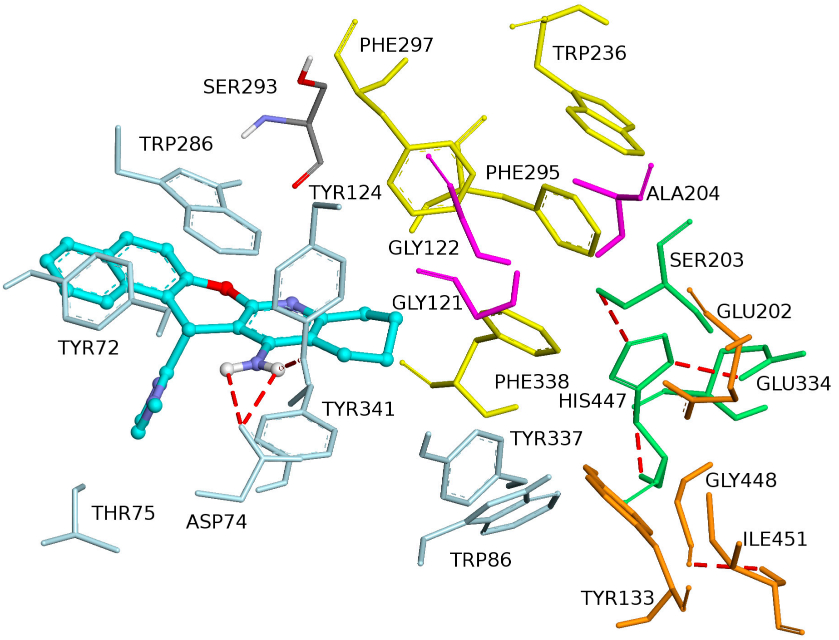

Figure S1. Binding mode of inhibitor (*R*)-8 at the active site of *Ee*AChE. Ligand is rendered as balls and sticks and illustrated in blue. The side chains conformations of the mobile residues are illustrated in the same color light as the ligand. Different subsites of the active site were colored: catalytic triad (CT) in green, oxyanion hole (OH) in pink, anionic sub-site (AS) in orange, except Trp86, acyl binding pocket (ABP) in yellow, and PAS in blue. Red dashed lines are drawn among atoms involved in hydrogen bond interactions.

The most energetically favorable binding mode of compound (*R*)-**8** at the active site of *Ee*AChE is shown in figure S1. This ligand shows a binding energy of −12.2 kcal/mol, it is located in the PAS and no interactions with the catalytic active site (CAS) were found

The examination of the first shell of residues surrounding (*R*)-**8** reveals that the pyranotacrine moiety was well fitted in the hydrophobic pocket composed by Tyr72, Tyr124, Trp286, Ile294, Phe295 and Tyr341. It is stacked against the indole and the benzene rings of Trp286 and Tyr341, respectively, through π-π interactions. The naphthalene moiety showed T-shape interaction with Tyr72. Moreover, three hydrogen bonds involving the amino group were observed. Asp74-O is engaged in a bifurcated hydrogen bond with both N-H protons of the amino group. The third one is established with Tyr124.

The binding pose of ligand (*S*)-**8** based on the docking results is presented in Figure S2. This compound binds effectively to the PAS through hydrogen bonding interactions and π-π stacking interactions (predicted binding energy of −11.8 kcal/mol). It is able to bind in the PAS by face-to-face π-π interactions between the pyranotacrine moiety of the ligand and Tyr341 phenyl ring and the Trp286 indole ring, and edge-to-face π-π interactions between naphthalene moiety of the ligand and Tyr72 phenyl ring. A hydrogen bond between the nitrogen atom of the pyridine ring and Tyr124-OH is established. The amino group of the ligand forms hydrogen bonding to the oxygen of the carbonyl Tyr341. Consequently, it can be postulated that racemic compound **8,** due to its large size, is unable to enter completely into the narrow active site gorge of the AChE receptor and hence acts only as a PAS binding site, as a non-competitive inhibitor.

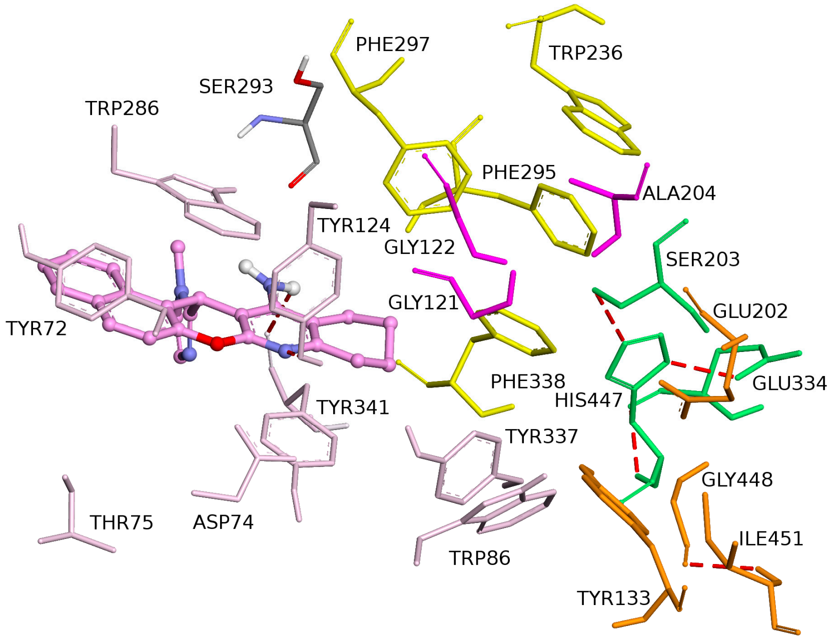

Figure S2. Binding mode of inhibitor (*S*)-**8** at the active site of *Ee*AChE. Ligand is rendered as balls and sticks and illustrated in pink. The side chains conformations of the mobile residues are illustrated in the same color light as the ligand. Different subsites of the active site were colored: CT in green, oxyanion hole (OH) in pink, AS in orange, except Trp86, ABP in yellow, and PAS in blue. Red dashed lines are drawn among atoms involved in hydrogen bond interactions.

Lastly, the binding modes overlay of compound **8** was examined. Both enantiomers share the position of cyclohexane and phenyl rings from which the molecules are arranged as mirror images (Figure S3).

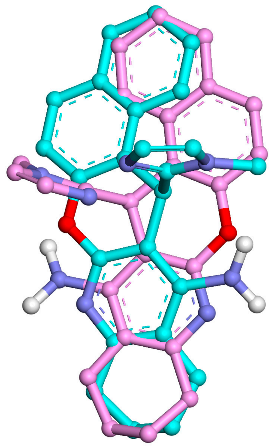

Figure S3. Overlay of binding mode of (*R*)-**8** (blue) and (*S*)-**8** (pink).

Next, we carried out the docking analysis of compound **8** on BuChE, in order to explain why this compound was a poor BuChE inhibitor.

In figure S4 we show the position of the top-scored poses of both enantiomers. The docking simulation of (*R*)-**8** and (*S*)-**8** shows that these compounds could not be accommodated efficiently inside the active site gorge, as the orientation of the rings does not allow penetration of molecules deep into the gorge, thereby, hindering their interactions with the amino acids in the active site.

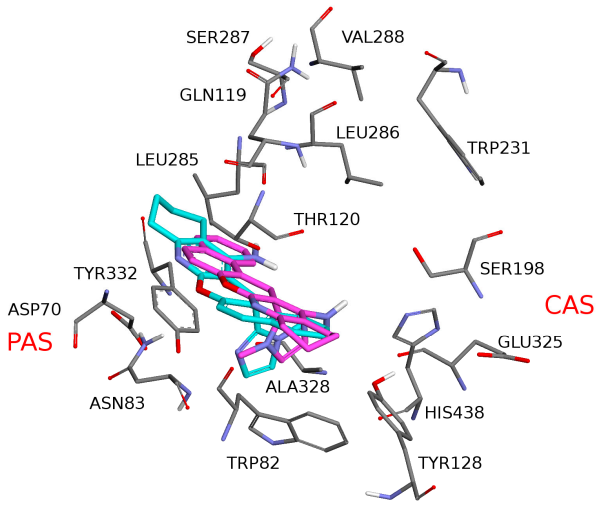

Figure S4. Complexes of compound 8 and eqBuChE homology built 3D-model. (*R*)-**8** is illustrated in blue and (*S*)-**8** in pink.

Molecular Modeling Methods

As the inhibitors were tested as racemic mixtures in the assay, both enantiomeric forms were built up and used for docking. (*R*)-**8** and (*S*)-**8** were assembled within Discovery Studio, version 2.1, software package, using standard bond lengths and bond angles. With the CHARMm force field [2], and partial atomic charges, the molecular geometries of (*R*)-**8** and (*S*)-**8** were energy-minimized using the adopted-based Newton-Raphson algorithm. Structures were considered fully optimized when the energy changes between iterations were less than 0.01 kcal/mol [3].

The coordinates of *Ee*AChE (PDB ID: 1C2B), were obtained from the Protein Data Bank (PDB). For docking studies, initial protein was prepared by removing all water molecules, heteroatoms, any co-crystallized solvent and the ligand. Proper bonds, bond orders, hybridization and charges were assigned using protein model tool in Discovery Studio, version 2.1, software package. CHARMm force field was applied using the receptor-ligand interactions tool in Discovery Studio, version 2.1, software package. Docking calculations were performed with the program Autodock Vina [1]. AutoDockTools (ADT; version 1.5.4) was used to add hydrogens and partial charges for proteins and ligands using Gasteiger charges. Flexible torsions in the ligands were assigned with the AutoTors module, and the acyclic dihedral angles were allowed to rotate freely. Trp286, Tyr124, Tyr337, Tyr72, Asp74, Thr75, Trp86, and Tyr341 receptor residues were selected to keep flexible during docking simulation using the AutoTors module. Because VINA uses rectangular boxes for the binding site, the box center was defined and the docking box was displayed using ADT. For *Electrophorus electricus* AChE (PDB ID: 1C2B) the docking procedure was applied to whole protein target, without imposing the binding site ("blind docking"). The search space was defined as a box of 60 × 60 × 72 with grid points separated 1 Å, which centered at the middle of the protein (x = 21.5911; y = 87.752; z = 23.591). The num_modes were set to 40 and the other parameters were left as default values. Finally, the most favorable conformations based on the free energy binding were selected for analyzing the interactions between the AChE and inhibitor. All the 3D models are depicted using Discovery Studio, version 2.1. The AutoDock Vina docking procedure used was previously validated [4,5].

The eqBuChE model has been retrieved from the SWISS-MODEL Repository [6–8]. This is a database of annotated three-dimensional comparative protein structure models generated by the fully automated homology-modeling pipeline SWISS-MODEL. A putative three-dimensional structure of eqBuChE has been created based on the crystal structure of *h*BuChE (PDB ID: 2PM8), these two enzymes exhibited 89% sequence identity. Initial protein was prepared and docking calculations were performed following the same protocol described before for *Ee*AChE. All dockings were performed as blinds dockings where a cube of 75 Å with grid points separated 1 Å, was positioned at the middle of the protein (x = 29.885; y = −54.992; z = 58.141). Default parameters were used except

num_modes, which was set to 40. The lowest docking-energy conformation was considered as the most stable orientation. Finally, the docking results generated were directly loaded into Discovery Studio, version 2.1.

2. ADMET of *Imidazopyranotacrines* 2, 4, 8 and 10

The Absorption Distribution Metabolism and Elimination (ADME) properties were calculated using the QikProp module of Schrodinger suite (QikProp, version 3.8, Schrodinger, LLC, New York, NY, 2013) running in normal mode, for assessing the druggability, and are shown in Tables S1 and S2.

Table S1. Physicochemical properties for compounds **2**, **4**, **8** and **10**.

Molecule	MW	SASA	Volume	DonorHB	AccptHB	QPlogPo/w	QPlogS
(*R*)-**2**	368.435	649.795	1.168.136	1.500	6.000	3.566	−5.429
(*S*)-**2**	368.435	642.089	1.153.418	1.500	6.000	3.548	−5.296
(*R*)-**4**	338.408	583.823	1.054.953	1.500	6.000	2.822	−4.469
(*S*)-**4**	338.408	579.749	1.043.199	1.500	6.000	2.854	−4.401
(*R*)-**8**	382.464	635.662	1.161.211	1.500	4.000	4.752	−6.174
(*S*)-**8**	382.464	620.575	1.142.369	1.500	4.000	4.735	−5.907
(*R*)-**10**	368.437	623.285	1.119.691	1.500	4.000	4.454	−5.955
(*S*)-**10**	368.437	618.353	1.115.842	1.500	4.000	4.518	−5.867

Table S2. Physicochemical properties for compounds **2**, **4**, **8** and **10**.

Molecule	QPPCaco	PSA	Metab	QPlogKhsa	QPlogBB	%HOA	ROF	ROT
(*R*)-**2**	1.429.814	86.525	5	0.490	−0.485	100	0	0
(*S*)-**2**	1.836.913	80.005	5	0.453	−0.367	100	0	0
(*R*)-**4**	1.172.872	83.200	5	0.259	−0.441	100	0	0
(*S*)-**4**	1.685.211	76.680	5	0.229	−0.292	100	0	0
(*R*)-**8**	2.430.908	54.593	4	0.934	−0.104	100	0	1
(*S*)-**8**	3.376.620	48.109	4	0.887	−0.050	100	0	1
(*R*)-**10**	2.158.639	56.422	4	0.820	−0.161	100	0	1
(*S*)-**10**	2.793.990	48.748	4	0.811	−0.043	100	0	1

MW: Molecular weight of the molecule (130.0–725.0).
SASA: Total Solvent Accessible Surface Area, in square angstroms, using a probe with a 1.4 Å radius (limits 300.0–1000.0).
volume: Total solvent-accessible volume, in cubic angstroms, using a probe with a 1.4 Å radius (limits 500.0–2000.0).
donorHB: Estimated number of hydrogen bonds that would be accepted by the solute (limits: 2.0–20.0).
accptHB: Estimated number of hydrogen bonds that would be donated by the solute (limits: 0.0-6.0).
QPlogPo/w: Predicted octanol/water partition coefficient (limits −2.0–6.5).
QPlogS: Predicted aqueous solubility. S, in mol/dm^3, is the concentration of the solute's saturated solution that is in equilibrium with crystalline solid (limits −6.5–0.5).
QPPCaco: Predicted apparent Caco-2 cell permeability in nm/sec. Caco-2 cells are a model for the gut-blood barrier.
QikProp predictions are for non-active transport. (<25 poor, >500 great).
PSA: Van der Waals surface area of polar nitrogen and oxygen atoms (limits 7.0-200.0). QPlog BB-Predicted brain/blood partition coefficient (limits -3.0–1.2).
metab: Number of likely metabolic reactions (limits 1–8).
QPlogKhsa: Prediction of binding to human serum albumin (limits −1.5-1.5).
HOA: Predicted qualitative Human Oral Absorption on 0 to 100% scale.
ROF: Number of violations of Lipinski's Rule of Five [9]. (molecular weight < 500, QPlogPo/w < 5, number of hydrogen bond donor ≤ 5, number of hydrogen bond acceptors HB ≤ 10).
ROT: Number of violations of Jorgensen's rule of three [10,11] (QPlogS> -5.7, QPCaco> 22 nm/s, number of primary metabolites < 7).

References

1. Trott, O.; Olson, A.J. AutoDock Vina: Improving the speed and accuracy of docking with a new scoring function, efficient optimization, and multithreading. *J. Comput. Chem*. **2010**, *31*, 455–461.
2. Brooks, B.R.; Bruccoleri, R.E.; Olafson, B.D.; States, D.J.; Swaminathan, S.; Karplus, M. A program for macromolecular energy, minimization, and dynamics calculations. *J. Comput. Chem*. **1983**, *4*, 187–217.
3. Morreale, A.; Maseras, F.; Iriepa, I.; Gálvez, E. Ligand-receptor interaction at the neural nicotinic acetylcholine binding site: A theoretical model. *J. Mol. Graph. Model*. **2002**, *2*, 111–118.
4. Bartolini, M.; Pistolozzi, P.; Andrisano, V.; Egea, J.; López, M.G.; Iriepa, I.; Moraleda, I.; Gálvez, E.; Marco-Contelles, J.L.; Samadi, A. *Chem. Med. Chem.* **2011**, *6*, 1990–1997.
5. Biasini, M.; Bienert, S.; Waterhouse, A.; Arnold, K.; Studer, G.; Schmidt, T.; Kiefer, F.; Tiziano Cassarino, G.; Bertoni, M.; Bordoli, L.; *et al*. SWISS-MODEL: Modelling protein tertiary and quaternary structure using evolutionary information. *Nucleic Acids Res.* **2014**, *42* (W1): W252–W258.
6. Arnold, K.; Bordoli, L.; Kopp, J.; Schwede, T. The SWISS-MODEL Workspace: A web-based environment for protein structure homology modelling. *Bioinformatics* **2006**, *22*, 195–201.
7. Kiefer F.; Arnold K.; Künzli M.; Bordoli L.; Schwede T. The SWISS-MODEL Repository and associated resources. *Nucleic Acids Res.* **2009**, *37*, D387–D392.
8. Guex, N.; Peitsch, M.C.; Schwede, T. Automated comparative protein structure modeling with SWISS-MODEL and Swiss-PdbViewer: A historical perspective. *Electrophoresis* **2009**, *30*, S162–S173.
9. Lipinski, C.A.; Lombardo, F.; Dominy, B.W.; Feeney, P.J. Experimental and computational approaches to estimate solubility and permeability in drug discovery and development settings. *Adv. Drug Deliv. Rev.* **2001**, 46, 3–26.
10. Duffy, E.M.; Jorgensen, W.L. Prediction of Properties from Simulations: Free Energies of Solvation in Hexadecane, Octanol, and Water. *J. Am. Chem. Soc.* **2000**, *122*, 2878–2888.
11. Jorgensen, W.L.; Duffy, E.M. Prediction of Drug Solubility from Monte Carlo Simulation. *Bioorg. Med. Chem. Lett*. **2000**, *10*, 1155–1158.
